# Alternating 2D and 3D culture reduces cell size and extends the lifespan of placenta-derived mesenchymal stem cells

**DOI:** 10.3389/fbioe.2025.1632810

**Published:** 2025-08-22

**Authors:** Ying Pan, Li Han, Yakun Yang, Xinran Wu, Aijun Wang, Liangqi Xie, Wuqiang Zhu, Shue Wang, Yuguo Lei

**Affiliations:** ^1^ Department of Biomedical Engineering, Pennsylvania State University, University Park, PA, United States; ^2^ Huck Institutes of Life Sciences, Pennsylvania State University, University Park, PA, United States; ^3^ Department of Biomedical Engineering, University of California, Davis, CA, United States; ^4^ Department of Surgery, Center for Surgical Bioengineering, School of Medicine, University of California, Davis, Sacramento, CA, United States; ^5^ Institute for Pediatric Regenerative Medicine, Shriners Hospitals for Children, Sacramento, CA, United States; ^6^ Cancer Biology and Infection Biology, Lerner Research Institute, Cleveland Clinic, Cleveland, OH, United States; ^7^ Department of Cardiovascular Medicine, Physiology and Biomedical Engineering, Center for Regenerative Medicine, Mayo Clinic Arizona, Scottsdale, AZ, United States; ^8^ Department of Chemistry, Chemical and Biomedical Engineering, University of New Haven, West Haven, CT, United States

**Keywords:** mesenchymal stem cells, alternating 2D/3D culture, spheroid culture, cell size, senescence

## Abstract

**Background:**

Mesenchymal stem cells (MSCs) hold great promise for treating a variety of human diseases; however, their clinical translation is hindered by challenges in large‐scale expansion while preserving therapeutic potency and maintaining small cell size. Conventional 2D culture on rigid substrates induces MSC senescence and enlargement, compromising their function and biodistribution.

**Methods:**

We present an alternating 2D/3D culture strategy that combines adherent monolayer expansion with transient spheroid formation to mitigate these limitations. Placenta‐derived MSCs were cultured under optimized spheroid conditions, with extracellular matrix supplementation and chemically defined media to enhance viability. To address scalability, we developed RGD-functionalized alginate hydrogel tubes (AlgTubes) that enable dynamic transitions between adherent and spheroid states for continuous culture.

**Results:**

Spheroid culture significantly reduced cell size and enhanced immunomodulatory function. The alternating 2D/3D protocol slowed MSC enlargement and senescence over multiple passages while preserving anti-inflammatory activity. Extracellular matrix supplementation and chemically defined media further improved cell viability. AlgTubes successfully supported the alternating culture strategy in a continuous and scalable format.

**Conclusions:**

The alternating 2D/3D culture system effectively overcomes limitations of conventional MSC expansion by mitigating enlargement, delaying senescence, and preserving both proliferative capacity and immunoregulatory potency. Combined with AlgTube technology, this work demonstrates a promising strategy for MSC manufacturing

## Introduction

Mesenchymal stem cells (MSCs) are multipotent stromal cells characterized by their capacity for self-renewal and differentiation into various mesenchymal lineages, including osteoblasts, chondrocytes, and adipocytes (Pittenger et al., 2019). Initially identified in the bone marrow, MSCs have since been isolated from diverse tissues, including adipose tissue, umbilical cord, dental pulp, and placenta (Pittenger et al., 2019). MSCs have garnered significant attention as therapeutics due to their exceptional safety profile and wide-ranging functions, including enhancing tissue repair, promoting angiogenesis, reducing fibrosis, cytoprotection, anti-inflammation, neutralizing reactive oxygen species (ROS), inhibiting NETosis, suppressing T cell activation, promoting Treg differentiation, and polarizing M2 macrophages (Pittenger et al., 2019). For instance, MSCs have demonstrated the capability to reduce infarct size and improve cardiac function following myocardial infarction in animal models. MSC transplantation enhances angiogenesis, reduces fibrosis, and promotes cardiomyocyte survival (Lim et al., 2018; Shake et al., 2002; Berry et al., 2006; Luger et al., 2017; Shafei et al., 2017; Dong et al., 2012; Shyu et al., 2006; Bagno et al., 2018). In experimental autoimmune encephalomyelitis (EAE) mice, MSCs reduce central nervous system inflammation and enhance neurological recovery (Yousefi et al., 2013; Lei et al., 2014; Rafei et al., 2009; Wang et al., 2016; Al Jumah and Abumaree, 2012; Morando et al., 2012). In rabbit and dog models of osteoarthritis, MSCs reduce cartilage degradation, alleviate pain, and improve joint function (Chiang et al., 2016; Riester et al., 2017; Deng et al., 2024; Kim H. et al., 2019; Kim S. E. et al., 2019; Maki et al., 2020; Zhang et al., 2018). As of March 2025, more than 1,800 clinical studies involving MSCs and their secretome have been registered on clinicaltrials.gov, targeting over 920 medical conditions such as Acute Respiratory Distress Syndrome (ARDS), sepsis, Graft-versus-Host Disease (GvHD), stroke, spinal cord injury, myocardial infarction, multiple sclerosis, organ transplantation, rheumatoid arthritis, Crohn’s, systemic lupus erythematosus, ulcerative colitis and COVID-19 (Le Blanc et al., 2008; Karussis et al., 2010; He et al., 2018; McIntyre et al., 2018; Chen et al., 2020; Connick et al., 2012; Wilson et al., 2015; Panés et al., 2016; Kebriaei et al., 2020; Zheng et al., 2014; Lv et al., 2020; Matthay et al., 2019; Gennadiy et al., 2018). A meta-analysis of 55 randomized clinical studies involving 2,696 patients finds that MSCs do not induce significant adverse effects (Thompson et al., 2020). No tumorgenicity and pro-coagulation risks are found (Thompson et al., 2020). Thirteen MSC-based therapies have been approved for clinical use worldwide.

However, MSC therapies face a significant challenge - the difficulty of producing large quantities of MSCs while preserving their functions and maintaining a small cell size. Currently, MSCs are expanded as a monolayer on rigid polymer substrates, including plastic flasks, polymer microcarriers in stirred tank bioreactors (STR), polymer scaffolds in packed bed (PB) bioreactors, and polymer hollow fibers in hollow fiber (HF) bioreactors (Mizukami et al., 2018; Amanda Mizukami and Swiech, 2018; Roh et al., 2016; Schnitzler et al., 2016). *In vivo*, MSCs reside in a soft, three-dimensional (3D) niche rich in cell-cell and cell-matrix interactions, as well as autocrine and paracrine signaling (Pittenger et al., 2019). Current cell culture technologies provide a starkly different two-dimensional (2D) stiff microenvironment. For instance, plastic flasks have a Young’s modulus of ∼100,000 kPa, which is far stiffer than natural soft tissues (Skardal et al., 2013; Huebsch, 2019). Using current methods, MSCs rapidly undergo senescence, losing their replicative ability and therapeutic potency (Yang et al., 2018; Kureel et al., 2019). This may explain the discrepancy between compelling preclinical data and less effective clinical outcomes (Matthay et al., 2019; Chen et al., 2016; Weiss, 2019; Galipeau, 2013). While preclinical studies use young, potent MSCs, clinical trials often rely on high-passage MSCs with impaired proliferation and functions.

MSCs also enlarge during *in vitro* expansion (Mo et al., 2018; Ge et al., 2014; Lu et al., 2024; Ji et al., 2024). A critical determinant of MSC therapeutic efficacy is their *in vivo* biodistribution. After systemic administration, MSCs frequently encounter a “first-pass effect,” with most cells trapped in organs such as the lungs, liver, and kidney (Zhuang et al., 2021; Sanchez-Diaz et al., 2021). Cell size significantly influences this process; larger MSCs are more likely to become lodged in the microvasculature of these organs, impairing their ability to reach target tissues (Ji et al., 2024). Moreover, oversized MSCs may cause microcirculation obstruction, ischemia, or stroke (Ge et al., 2014; Guo et al., 2014; Janowski et al., 2013). There is a need for new culture strategies that can efficiently expand MSCs without causing cell enlargement and loss of function.

Research finds that culturing MSCs as spheroids (referred to as spheroid culture) may overcome some of these limitations (Cesarz and Tamama, 2016; Hazrati et al., 2022; Ohori-Morita et al., 2025). Spheroid Culture has been shown to mitigate senescence, preserving a youthful phenotype with smaller cell size, improved survival, increased secretion of trophic factors, and elevated expression of stemness-related genes (Mo et al., 2018; Lu et al., 2024; Bijonowski et al., 2020a; Bijonowski et al., 2020b). However, due to their anchorage-dependent nature, MSCs do not proliferate effectively in spheroid culture, limiting their utility for large-scale MSC expansion. In this work, we present an approach that combines the benefits of both 2D and 3D spheroid culture methods to grow placenta-derived MSCs. Briefly, MSCs are expanded as adherent monolayers in 2D flasks for several days. After each passage, MSCs are transitioned to a non-adherent environment for 24–72 h to form 3D spheroids. For simplicity, we refer to this method as the “alternating 2D/3D culture protocol.” We hypothesize that spheroid formation following 2D expansion can restore MSC size and function, thereby mitigating cell senescence and enlargement.

## Methods

### MSC isolation

**FIGURE 1:**
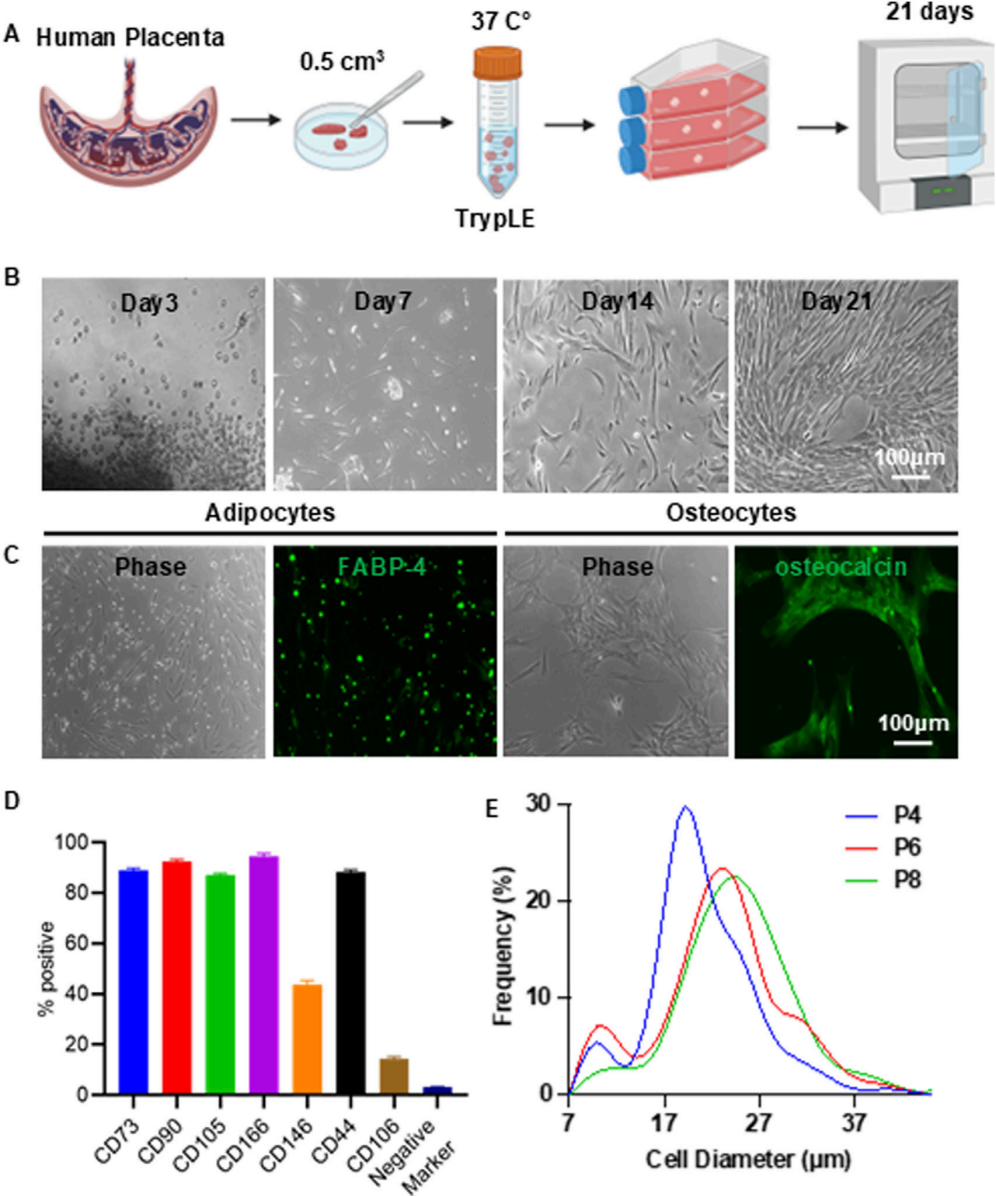
Isolation of Placenta-Derived MSCs. **(A)** Illustration of the MSC isolation process. **(B)** Representative morphology of MSCs during isolation. **(C)** Differentiation of MSCs into adipocytes (FABP-4^+^) and osteocytes (osteocalcin^+^). **(D)** Flow cytometry analysis of MSC surface markers. Negative markers include CD34, CD45, CD11b, CD79A, and HLA-DR. **(E)** Size distribution of MSCs cultured in T-25 flasks at passages 4 (P4), 6 (P6), and 8 (P8).

Full-term human placentas were obtained from ZenBio Inc. Briefly, placentas were washed and cut into approximately 0.5 cm^3^ pieces, which were then partially digested with TrypLE Select solution (Gibco) at 37 °C for 30 min (Figure 1A). Following digestion, 15–20 tissue pieces were transferred to a 75 cm^2^ tissue culture flask containing 9 mL of EBM-2 complete medium (EBM-2 supplemented with 10% fetal bovine serum and 1% Penicillin-Streptomycin). The flasks were placed in an incubator and left undisturbed for 3 days to allow the tissue to attach. After this period, the medium was replaced every 3 days until the cells reached confluence. These cells were designated as passage 0 (P0). P0 cells were either cryopreserved or subcultured. Details of MSC isolation and characterization can be found in our previous publication (Han et al., 2023).

### MSC surface marker characterization

MSCs were characterized using the Human Mesenchymal Stem Cell Verification Flow Kit (R&D Systems), which includes antibodies against the positive markers CD90, CD73, and CD105, as well as the negative markers CD45, CD34, CD11b, CD79A, and HLA-DR. Additionally, the Human Mesenchymal Stem Cells Multi-Color Flow Kit (R&D Systems) was used to assess expression of the positive markers CD44, CD106, CD146, and CD166. Flow cytometric analysis was performed using the BD FACSCanto™ II System.

### MSC differentiation

MSCs were evaluated for their differentiation potential using the Human Mesenchymal Stem Cell Functional Identification Kit (R&D Systems), following the manufacturer’s instructions. After 21 days of induction, cells were fixed and stained with an FABP-4 antibody to identify adipocytes and an osteocalcin antibody to identify osteocytes.

### MSC 2D culture

MSCs were seeded into T-25 flasks at a density of 8,000 cells/cm^2^ in 4 mL of EBM-2 complete medium. The culture medium was replaced every 3 days. Once the cells reached approximately 90% confluency, they were harvested with 0.25% Trypsin and sub-cultured at the same seeding density.

### MSC 3D spheroid culture

Dissociated single MSCs were seeded into 96-well clear, round-bottom, ultra-low attachment microplates (Corning) at densities ranging from 2,000 to 40,000 cells per well in 200 μL of EBM-2 complete medium or other indicated media. The medium was refreshed every 3 days. To promote spheroid formation, plates were centrifuged at 100 *g* for 8 min. Spheroids were cultured for the indicated duration. To assess cell viability, propidium iodide was added to the medium and incubated for 30 min before imaging with fluorescence microscopy.

### Alternating 2D/3D culture

MSCs harvested from 2D culture (25,000 cells per well) were seeded into 96-well clear, round-bottom, ultra-low attachment microplates to form spheroids. After 48 h, the spheroids were dissociated into single cells, which were then seeded into 2D T-25 flasks at a density of 8,000 cells/cm^2^ in 4 mL of EBM-2 complete medium. The medium was changed every 3 days. Once the cells reached approximately 90% confluency, they were harvested and cultured again as spheroids for 48 h before being replated onto 2D flasks.

### MSC size quantification

Spheroids were harvested and washed twice with PBS. To dissociate them into single cells, 2 mL of 0.25% trypsin-EDTA (Corning) was added and incubated at 37 °C in a water bath for 10 min. The enzymatic reaction was stopped by adding 2 mL of EBM-2 complete medium. Cells were then centrifuged at 300 *g* for 8 min and resuspended in EBM-2 complete medium. Images of the single cells were captured using a Zeiss fluorescence microscope. Cell size was analyzed using ImageJ software using the following procedure (Supplementary Figure S1):

• Import phase contrast images into ImageJ
• Set the image scale via Analyze → Set Scale
• Adjust threshold using Image → Adjust → Color Threshold
• Measure cell area using Analyze → Analyze Particles, setting the circularity range to 0.70–1.00
• Export the results to Excel and calculate cell diameter using the formula:

$$R=2\times \sqrt{\frac{S}{\pi }}$$

R: Cell diameter; S: Area value from Image.

### β-Galactosidase activity assay

Senescence-associated β-galactosidase activity was assessed using the CellEvent Senescence Green Detection Kit (Thermo Fisher Scientific), according to the manufacturer’s instructions. Cells cultured in 2D or alternating 2D/3D spheroid conditions were dissociated into single-cell suspensions and seeded into flat-bottom 96-well plates, allowing them to adhere overnight. Before staining, cells were washed with PBS and fixed with 2% paraformaldehyde (PFA) in PBS for 10 min at room temperature, protected from light. After an additional PBS wash containing 1% BSA, the CellEvent™ Working Solution was prepared by diluting the Senescence Green Probe (1:1,000) into pre-warmed (37 °C) Senescence Buffer and added to each well (100 µL/well). Plates were sealed and incubated for 2 h at 37 °C in the absence of CO_2_, protected from light. Following incubation, wells were washed three times with PBS, counterstained with DAPI, and imaged using a fluorescence microscope.

### Macrophage culture

RAW 264.7 cells (RAW-Dual™ cells, InvivoGen) were cultured in DMEM supplemented with 4.5 g/L glucose, 2 mM L-glutamine, 10% heat-inactivated fetal bovine serum (FBS), 100 µg/mL Normocin, and 1% Penicillin-Streptomycin. Cells were seeded at a density of 1.5 × 10^4^ cells/cm^2^, and the medium was replaced twice a week.

### Macrophage inflammation assay

RAW 264.7 macrophage cells were stimulated in DMEM complete medium containing 100 ng/mL lipopolysaccharide (LPS; O111:B4, Sigma) and 10 ng/mL murine interferon-gamma (IFN-γ; Peprotech). MSCs were co-cultured with RAW 264.7 cells at a ratio of 1:10 MSC/RAW. After 24 h, conditioned media were collected for cytokine analysis using enzyme-linked immunosorbent assays (ELISA). Additionally, the RAW-Dual cells are engineered to express a luciferase gene under the control of an ISG54 minimal promoter, in conjunction with five IFN-stimulated response elements (ISREs). It reports the activation of interferon regulatory factors (IRFs), which contribute to the inflammatory response. The luciferase level is measured using the commercial kit.

### Modifying alginates with RGD peptides

A 2% (w/v) alginate solution (Cat. #194-13321, 80–120 cP, Wako Chemicals) was prepared by dissolving alginate in 0.1 N NaOH. The solution was then reacted with divinyl sulfone (DVS) at a 1:3 M ratio of hydroxyl groups to DVS for 15 min. Excess DVS was removed by dialysis. Approximately 20%–30% of the hydroxyl groups in the alginate polymer were successfully modified with DVS. To synthesize alginate-RGD, RGD peptides containing a C-terminal cysteine were conjugated to the DVS-modified alginate under alkaline conditions. About 10% of the modified hydroxyl groups were functionalized with RGD peptides. The resulting alginate-RGD was then blended with unmodified alginate to prepare a 2% (w/v) alginate solution, which was used to fabricate alginate hydrogel tubes.

### Processing alginate hydrogel tubes (AlgTubes)

A custom-made micro-extruder was used to process AlgTubes. A hyaluronic acid (HA) solution containing single cells and an alginate/alginate-RGD solution were pumped into the central and side channels of the micro-extruder, respectively, to form coaxial core-shell flows that were extruded into a CaCl_2_ buffer (100 mM) to form AlgTubes. If necessary, AlgTubes were further soaked in 1 mM Poly (ethylene glycol) dithiol (HS-PEG-SH, Mw 3400) for 10 min at pH 8.0 to achieve secondary covalent crosslinking through the Michael addition reaction between -SH and -VS. Detailed methods for processing AlgTubes are described in our previous publications (Wang and Lei, 2019; Wang et al., 2021; Yang et al., 2025; Lin et al., 2019a; Li et al., 2018a; Lin et al., 2018a; Lin et al., 2019b; Liu et al., 2023; Li et al., 2018b; Lin et al., 2018b; Lin et al., 2019c).

### Culturing cells in AlgTubes

For standard cell culture, 20 µL of cell-laden AlgTubes were suspended in 2 mL of culture medium in each well of a 6-well plate. Cells were seeded at a density of 1–2 × 10^6^ cells/mL within the hydrogel tube space. The tubes were formed using 2% alginate modified with 1 mM RGD peptide. The resulting hydrogel tubes had diameters ranging from 200 to 300 μm, with shell thicknesses of approximately 30–70 µm. To detach MSC from the AlgTubes, 1.2 mM free RGD peptides were added to the culture medium.

### Statistical analysis

Experiments were performed in triplicate and repeated with MSCs from two donors. Representative data are presented in the Results section. Data were analyzed using GraphPad Prism 8 statistical software. P value was determined by one-way analysis of variance (ANOVA) for comparison between the means of three or more groups or unpaired two-tailed t-tests for two-group analysis. The significance levels are indicated by p-value, *: p < 0.05, **: p < 0.01, ***: p < 0.001.

## Results

### Isolate and characterize placenta-derived MSCs

Full-term placental tissue was minced into small fragments and enzymatically digested with TrypLE for 30 min. The digested tissue was transferred to a T-25 cell culture flask and incubated undisturbed for 3 days to allow the fragments to adhere to the flask surface (Figure 1A). By day 3, cells began migrating out of the tissue explants. By day 7, numerous spindle shape MSCs were visible. By day 21, the culture had expanded significantly, covering approximately 70% of the flask surface (Figure 1B). At this stage, the remaining tissue fragments were removed, and the adherent cells were expanded to full confluence. Cells were harvested using 0.25% trypsin and either cryopreserved or subcultured.

The isolated cells exhibited the characteristic spindle shape morphology of MSCs. They exhibited multipotency, as demonstrated by their ability to differentiate into FABP4-positive adipocytes and osteocalcin-positive osteocytes (Figure 1C). Flow cytometry analysis revealed that most cells expressed MSC surface markers, including CD73, CD90, CD105, CD44, and CD166. In contrast, expression of hematopoietic and immune markers—such as CD45, CD34, CD11b, CD79A, and HLA-DR—was negligible (Figure 1D; Supplementary Figure S2). In our previous study, we demonstrated that these cells possess strong immunomodulatory capabilities, as they suppress cytokine release from macrophages, inhibit reactive oxygen species (ROS) production and neutrophil extracellular trap (NET) formation by neutrophils, and reduce T cell proliferation under inflammatory conditions *in vitro* (Han et al., 2023). In an acute lung injury mouse model, these MSCs significantly lowered cytokine levels, reduced tissue damage, and improved survival outcomes (Han et al., 2023). When these MSCs were cultured over multiple passages, a clear trend of increasing cell size with successive passages was observed (Figure 1E). This observation is consistent with previous reports that MSCs tend to enlarge during *in vitro* Culture on stiff 2D surfaces.

### Optimize spheroid culture condition

**FIGURE 2:**
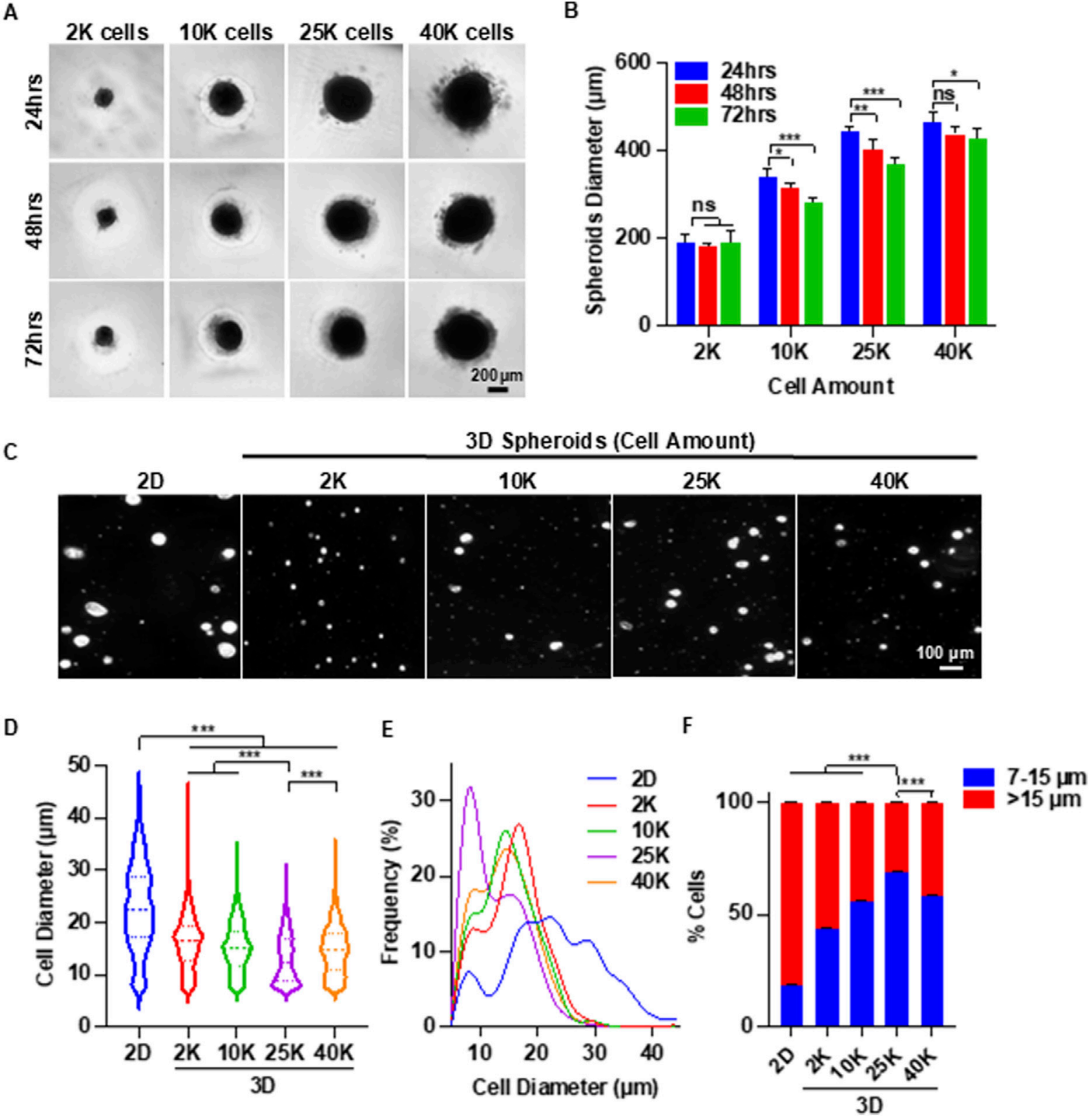
3D Spheroid Culture Reduces MSC Size: Identifying the Optimal Spheroid Diameter. **(A)** Representative images of MSC (P3) spheroids cultured for 3 days with varying initial cell numbers per spheroid. **(B)** Quantification of spheroid diameter over the 3-day culture period. **(C)** Representative images of individual MSCs following 2D and 3D spheroid culture. **(D,E)** Cell size distribution of MSCs after 2D and 3D spheroid culture. **(F)** Proportions of small (≤15 µm) and large (>15 µm) MSCs after 2D and 3D spheroid culture. Spheroid culture duration in **(C–F)** was 72 h.

We first investigated whether placenta MSCs could form spheroids. MSCs were seeded into low attachment 96-well plates at densities of 2,000, 10,000, 25,000, and 40,000 cells per well. All groups successfully formed spheroids within 24 h (Figure 2A). At this time point, the average diameters of the spheroids were 191.0 µm for 2 K, 338.1 µm for 10 K, 443.1 µm for 25 K, and 462.7 µm for 40 K. Notably, the 2 K spheroids maintained a consistent diameter over extended culture periods (48 and 72 h), whereas the diameters of the 10, 25, and 40 K spheroids decreased with prolonged Culture (Figure 2B).

Next, we assessed whether spheroid culture could reduce the size of individual MSCs. Spheroids were dissociated into single cells and imaged using phase-contrast microscopy. Regardless of the spheroid size, MSCs derived from spheroids appeared markedly smaller than those from 2D cultures (Figure 2C). Using ImageJ software, we measured the diameters of over 600 MSCs per group following a standardized protocol (Supplementary Figure S1). The mean diameter of MSCs from 2D culture was 22.8 µm. MSCs from 2, 10, 25, and 40 K spheroids cultured for 72 h had mean diameters of 16.1, 15.1, 13.0, and 14.6 µm, respectively (Figure 2D). Additionally, MSCs from 2D culture exhibited a much broader size distribution compared to those from spheroid cultures.

Interestingly, size distribution histograms revealed two distinct peaks (Figure 2E). The proportion of cells in the smaller size peak was significantly higher in 3D spheroid cultures than in 2D cultures. We categorized MSCs based on diameter: cells between 7 and 15 µm were defined as “small MSCs,” while those larger than 15 µm were classified as “big MSCs.” Small MSCs comprised less than 20% of the population in 2D cultures (Figure 2F). In contrast, at least 40% of MSCs are small in spheroid culture. Among the 3D groups, the 25 K spheroids yielded the highest proportion of small MSCs (∼70%). Therefore, 25 K spheroids were used in subsequent experiments.

**FIGURE 3:**
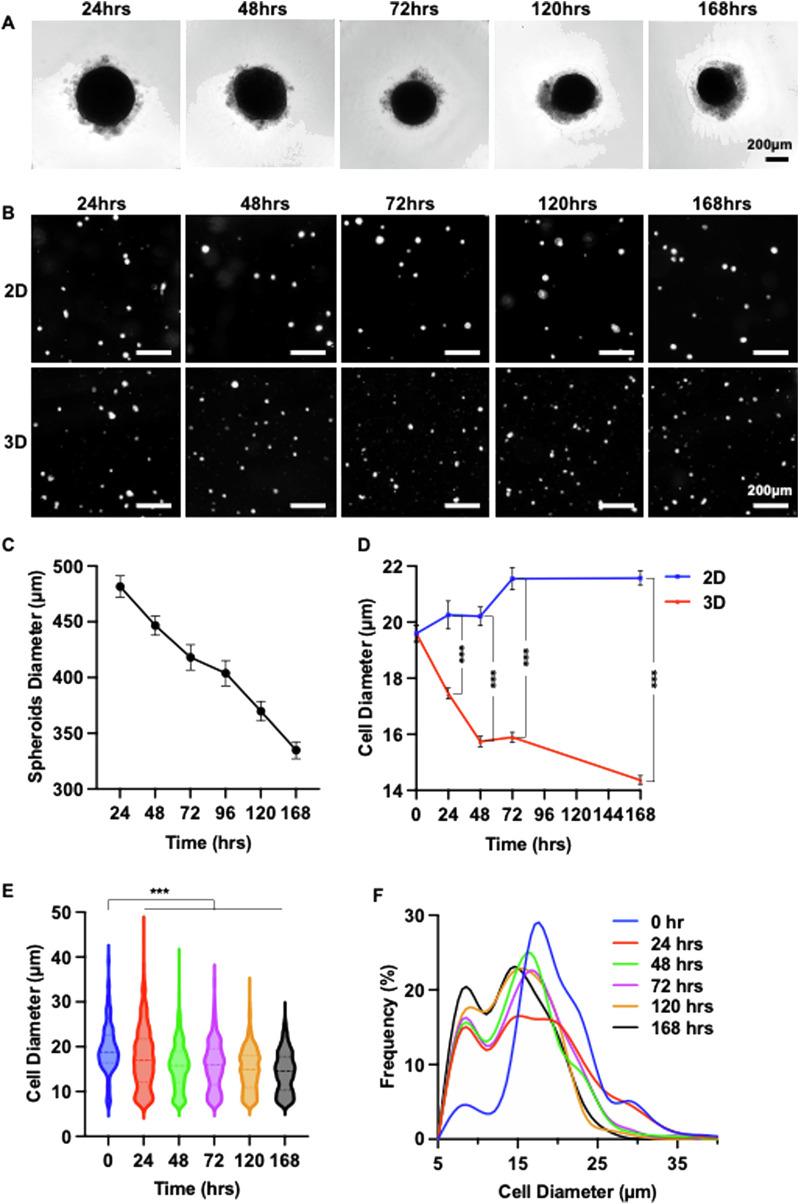
3D Spheroid Culture Reduces MSC Size: Determining the Optimal Culture Duration. **(A)** Representative images showing morphological changes in MSC (P4) spheroids over a 7-day culture period. **(B)** Images of individual MSCs cultured in 2D or in 25 K-cell spheroids for varying durations. **(C)** Changes in the diameter of 25 K-cell MSC 3D spheroids over 7 days. **(D)** Comparison of mean MSC diameter in 2D versus 25 K spheroid cultures over 7 days **(E,F)** Size distribution of MSCs within 25 K spheroids across the 7-day culture period.

After identifying the optimal cell number per spheroid, we systematically assessed the effect of spheroid culture duration. Spheroids with 25 K cells were cultured for up to 7 days. The diameter of the spheroid gradually decreased over time (Figures 3A,C). Spheroids were dissociated daily to measure cell size (Figure 3B). In contrast to 2D cultures, where MSC size steadily increased, spheroid-cultured MSCs exhibited a significant size reduction within the first 48 h. Although the size continued to decrease from 48 to 168 h, the reduction rate was less pronounced (Figure 3D). The MSC size distribution of MSCs became progressively narrower with longer culture durations (Figures 3E,F). These findings suggest that a spheroid culture duration of 48–72 h is optimal.

### Enhance cell viability with extracellular matrices and chemically defined medium

**FIGURE 4:**
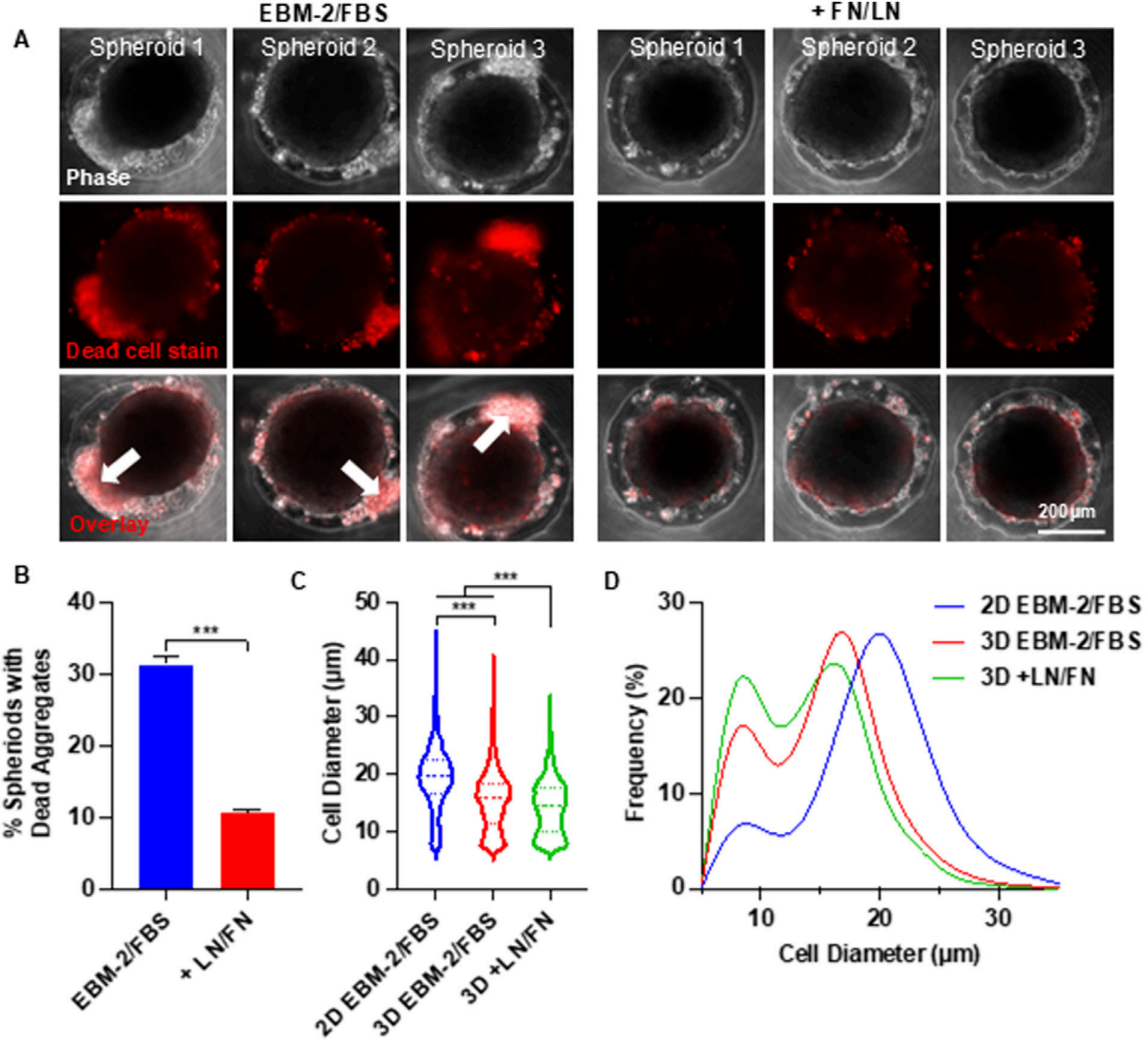
Effects of Supplementing Extracellular Matrix Proteins on MSC Viability and Size in Spheroid Culture. **(A)** Phase-contrast and dead cell staining (red) images of MSC (P4) spheroids cultured in EBM-2 + 10% FBS medium, with or without laminin (LN) and fibronectin (FN). Three representative spheroids are shown in each condition. The white arrow indicates the loosely associated cells. **(B)** Percentage of spheroids containing dead cell aggregates in the presence or absence of LN and FN. **(C,D)** MSC size distribution following 2D monolayer and 3D spheroid cultures. Spheroids were cultured for 48 h.

During the optimization experiments, we found that a significant portion of MSCs remained loosely attached to the core spheroids (Figure 4A). We stained the spheroids with Propidium Iodide (PI) and found these loosely associated cells were dead (Figure 4A). We tested whether supplementing extracellular matrix (ECM) proteins could mitigate cell death. MSCs were seeded at 25,000 cells per well in EBM-2 medium, with or without a mix of 0.625 µg/mL Laminin-511, 0.625 µg/mL Laminin-411, 0.625 µg/mL Laminin-521, and 6.25 µg/mL fibronectin. The ECM proteins significantly reduced the number of dead cells (Figure 4A). In the absence of ECM proteins, approximately 30% of spheroids exhibited dead cell aggregates. This number dropped to 10% with ECM supplementation (Figure 4B). ECM proteins also reduced the diameter of individual MSCs (Figure 4C) and increased the proportion of small MSCs (Figure 4D).

**FIGURE 5:**
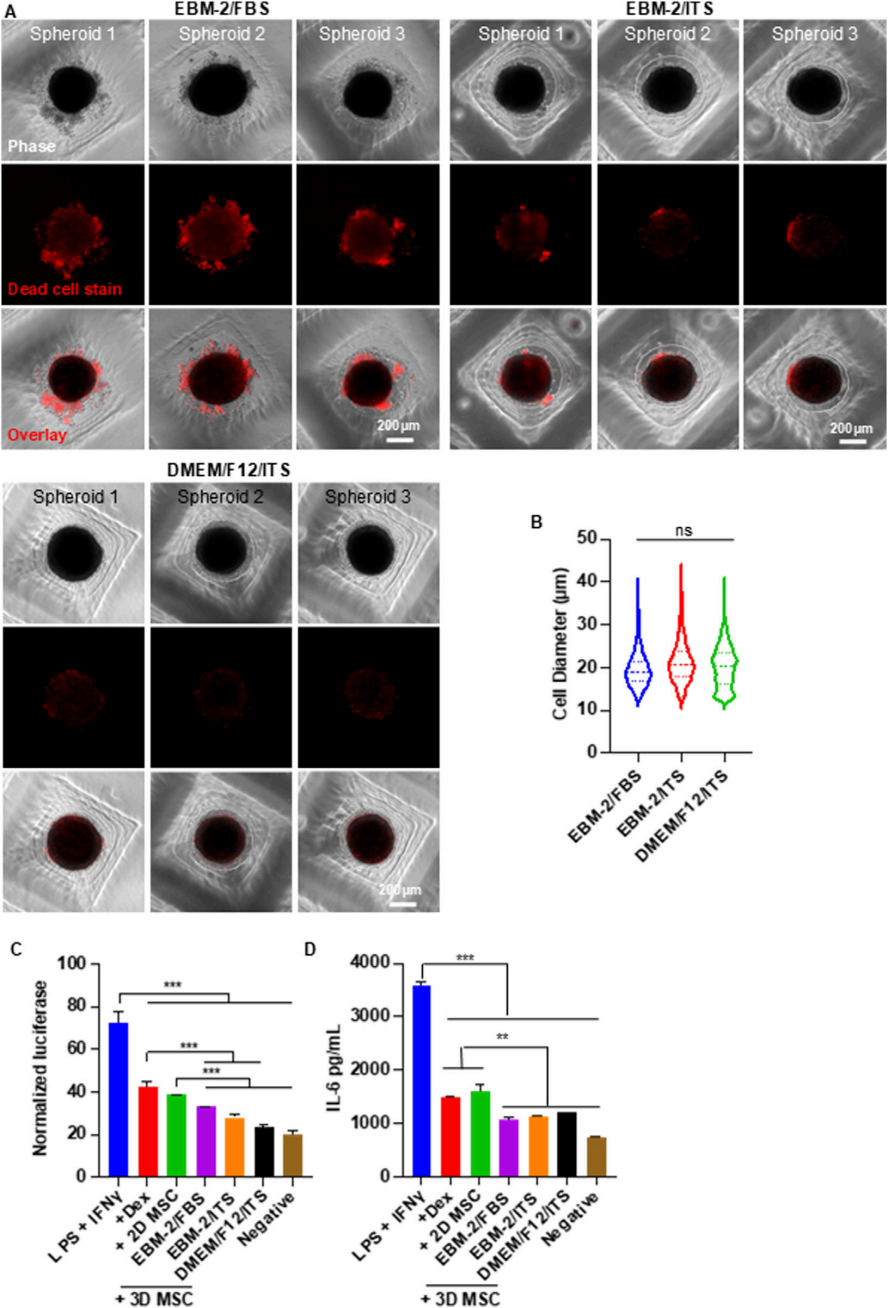
Effect of Chemically Defined Medium on MSC Viability and Size in Spheroid Culture. **(A)** Phase-contrast and dead cell staining (red) images of MSC (P6) spheroids cultured in various media. Three representative spheroids are shown in each condition. **(B)** MSC size distribution in 3D cultures across different media conditions. **(C,D)** Anti-inflammatory capability of MSCs cultured under varying conditions. RAW-Dual™ reporter cells were stimulated with 100 ng/mL LPS and 10 ng/mL IFNγ, then treated with MSCs. Dexamethasone (Dex, 1 µg/mL) was used as a positive control. Luciferase activity **(C)** and mouse IL-6 **(D)** were measured. Spheroid culture time was 48 h.

The medium used in the above experiments contained 10% fetal bovine serum (FBS). We further tested whether removing FBS could improve the cell viability. MSCs were seeded at 25,000 cells per well and cultured in three different media: EBM-2 basal medium + 10% FBS (EBM-2 + FBS), EBM-2 basal medium supplemented with insulin-transferrin-selenium (ITS) (EBM-2 + ITS), and DMEM/F12 basal medium supplemented with ITS (DMEM/F12 + ITS). The results confirmed a high number of dead cells in the FBS-containing medium (Figure 5A). Replacing FBS with ITS did not impair spheroid formation and significantly reduced cell death. Remarkably, spheroids cultured in DMEM/F12 + ITS exhibited almost no dead cells. The diameters of MSCs across the three media were comparable (Figure 5B).

To evaluate whether the defined medium affected MSC function, we assessed their ability to suppress macrophage-mediated inflammation (Figures 5C,D). We focused on the interferon regulatory factor (IRF) signaling pathway, a key regulator of inflammation. RAW 264.7 macrophages engineered to express a secreted luciferase reporter under IRF control were used. Cells were stimulated with 100 ng/mL LPS and 10 ng/mL IFNγ to induce inflammation. Inflamed macrophages were then treated with MSCs or Dexamethasone (1 µg/mL), a clinically used anti-inflammatory agent serving as a benchmark. After 24 h, the luciferase activity in the medium was quantified. The pro-inflammatory cytokine IL-6 was also measured using an ELISA assay, with antibodies specific to mouse IL-6 to minimize cross-reactivity with human cytokines secreted by MSCs. All treatments, including MSCs from both 2D and 3D cultures, reduced IL-6 and luciferase expression (Figures 5C,D). MSCs from 2D culture showed similar efficacy to dexamethasone, while MSCs from spheroid culture outperformed dexamethasone. No significant differences were observed among the three media conditions, indicating that chemically defined media do not compromise the immunomodulatory function of MSCs.

### Slows down MSC size enlargement and senescence via alternating 2D flask and 3D spheroid culture

**FIGURE 6:**
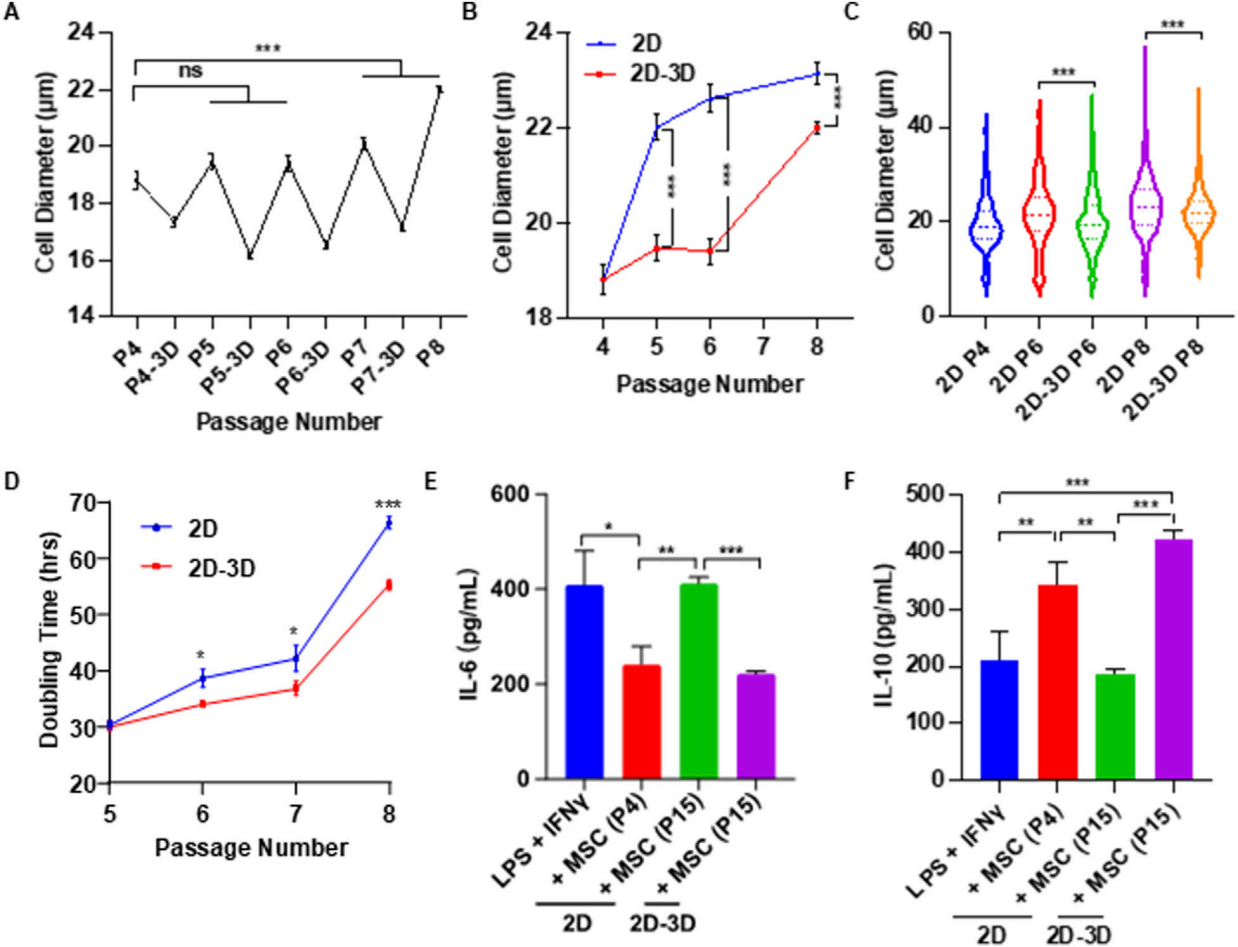
Effects of Alternating 2D/3D Culture on MSC Size and Immunomodulatory Function. **(A)** MSCs were cultured in flasks for four passages, with an additional 2-day spheroid culture following each passage. Shown are the mean cell diameters immediately after 2D culture and after subsequent spheroid culture. **(B)** MSC diameters from P5 to P8 using conventional 2D culture versus the alternating 2D/3D method. P4 cells served as the starting population for both conditions. Diameters were measured after harvesting from 2D flasks. **(C)** Comparison of MSC sizes at P5–P8 between the two culture methods. **(D)** Comparison of doubling times at P5–P8 between the two methods. **(E,F)** Macrophages were activated with LPS and IFNγ, then treated with either early-passage (P4) or late-passage (P15) MSCs derived from conventional 2D culture or the alternating 2D/3D protocol. Levels of mouse IL-6 and IL-10 were measured to assess immunomodulatory effects.

The above studies demonstrate that spheroid culture can reduce the size of MSCs and enhance their functions. However, anchorage-dependent MSCs do not proliferate in spheroids. To address this limitation, we tested a culture protocol that alternates between 2D and 3D environments—leveraging the proliferative capacity of 2D culture and the size-reducing, function-restoring benefits of 3D spheroid culture. Briefly, MSCs were expanded in standard 2D flasks. After each passage, cells were transferred to ultra-low attachment plates to form spheroids for 48 h (Figure 6A). We compared long-term culture outcomes between continuous 2D culture and the alternating 2D/3D protocol. Passage 4 MSCs were used as the starting materials. MSC diameter increased significantly after one passage (passage 5) in 2D Culture (Figure 6B). In contrast, MSCs cultured using the 2D/3D protocol maintained a smaller size until passage 8 (Figures 6B,C). At every passage, MSCs from the 2D/3D protocol were consistently smaller than those from 2D culture alone (Figure 6C). Additionally, the 2D/3D protocol resulted in shorter population doubling times, indicating that the spheroid culture partially restored the proliferative capability (Figure 6D).

To assess the impact of long-term Culture on MSC immunomodulatory function, cells were expanded to passage 15 (P15) using both methods. MSCs from 2D culture at passage 4 (2D-P4) and passage 15 (2D-P15), and 2D/3D culture at passage 15 (2D/3D-P15) were co-cultured with RAW 264.7 macrophages, and cytokine levels were measured by ELISA. For IL-6, a pro-inflammatory cytokine, secretion levels in the 2D-P4 and 2D/3D-P15 groups were comparable and significantly lower than those in the positive control group. In contrast, IL-6 levels in the 2D-P15 group were similar to those of the positive control, indicating a loss of immunosuppressive function (Figure 6E). For IL-10, an anti-inflammatory cytokine, 2D-P15 MSCs secreted levels comparable to those of the positive control, suggesting diminished function. 2D/3D-P15 MSCs secreted high levels of IL-10, similar to the 2D-P4 group, indicating preserved or enhanced immunomodulatory capacity (Figure 6F). We found most 2D-P15 MSCs became senescent, as shown by the high β-galactosidase (β-gal) activity (Supplementary Figure S3A). 2D/3D-P15 MSCs exhibited significantly less β-gal activity. Additionally, 2D/3D-P15 MSCs retained their differentiation potential, as shown by their ability to differentiate into adipocytes and osteocytes (Supplementary Figure S3B). These findings suggest that alternating 2D and 3D culture phases can effectively delay MSC enlargement and senescence while preserving their therapeutic potential.

## Discussion

In summary, our results show the potential of the alternating 2D/3D culture technique for MSC production. We demonstrated that placenta-derived MSCs were capable of forming spheroids, and that spheroid culture significantly reduced cell size (Figure 2). Both spheroid size and culture duration were found to influence MSC size (Figures 2, 3), consistent with previous reports in the literature (Cesarz and Tamama, 2016; Ohori-Morita et al., 2025; Sun et al., 2018; Fuentes et al., 2022). A notable proportion of MSCs underwent cell death during the formation of spheroids. We found that supplementing the Culture with ECM proteins such as laminins and fibronectins reduced this cell loss (Figure 4). Despite their effectiveness, these ECM proteins are costly and increase the overall production expense. We found that using a chemically defined and cost-effective medium - DMEM/F12 supplemented with ITS - not only prevented cell death, but also preserved MSC immunomodulation functions (Figure 5). This finding is particularly important for researchers developing MSC-derived extracellular vesicle (EV) therapeutics. Spheroid Culture has been shown to enhance EV yield, but a significant challenge in MSC EV manufacturing is the use of serum-containing media, which introduces contaminating EVs. Culturing spheroids in DMEM/F12 + ITS offers a serum-free and affordable alternative that mitigates this issue and could significantly advance the field of MSC EV-based medicine. Importantly, placenta-derived MSCs cultured using the alternating 2D/3D approach exhibited slower progression of cell enlargement and senescence. These cells also retained their immunomodulatory function, in contrast to their 2D-cultured counterparts, which lost this capability entirely (Figure 6).

### MSCs undergo enlargement and senescence in 2D culture

To meet the demand for large quantities of MSCs, they are typically expanded *in vitro* using 2D culture (Binato et al., 2013). However, culture in this artificial environment often leads to cell enlargement, loss of proliferation capability, and functions (Gu et al., 2016). Understanding the mechanisms behind these changes is essential for preserving MSC therapeutic efficacy (Binato et al., 2013). Research indicates that cellular senescence is a primary contributor to MSC enlargement. Like other somatic cells, MSCs have a finite replicative lifespan and enter senescence after undergoing repeated divisions (Gu et al., 2016). This process is characterized by a shift from the typical spindle shape morphology to an enlarged, flattened appearance (Gu et al., 2016). Senescent MSCs exhibit increased senescence-associated β-galactosidase (β-gal) activity and arrest in the G0/G1 phase (Gu et al., 2016). The cell cycle arrest, combined with continued biosynthetic activity, causes the cell to enlarge. Senescence can be driven by multiple interconnected processes (Weng et al., 2022; Roger et al., 2021; Neri and Borzì, 2020; Zhou et al., 2020), such as DNA damage accumulation, telomere shortening, mitochondrial dysfunction, impaired autophagy, and epigenetic alterations (Weng et al., 2022; Roger et al., 2021; Neri and Borzì, 2020; Zhou et al., 2020). DNA damage activates the DNA damage response (DDR) and downstream pathways, leading to cell cycle arrest (Weng et al., 2022; Roger et al., 2021; Neri and Borzì, 2020; Zhou et al., 2020). Similarly, telomere attrition from repeated replication triggers DDR and senescence (Weng et al., 2022; Roger et al., 2021; Neri and Borzì, 2020; Zhou et al., 2020). Mitochondrial dysfunction and defective autophagy result in the buildup of damaged cellular components, promoting senescence (Weng et al., 2022; Roger et al., 2021; Neri and Borzì, 2020; Zhou et al., 2020). Oxidative stress also plays a central role by inducing DNA damage and altering cell behavior (Gu et al., 2016; Weng et al., 2022; Roger et al., 2021; Neri and Borzì, 2020; Zhou et al., 2020).

In addition to intrinsic aging processes, the non-physiological conditions of 2D culture contribute to MSC enlargement. The rigid tissue culture plastic lacks the 3D architecture and mechanical cues of the native MSC niche (Cesarz and Tamama, 2016). Instead, it exposes cells to a flat, stiff surface, which alters morphology, gene expression, and function (Kouroupis and Correa, 2021). The stiffness of flasks far exceeds that of native tissues (typically <0.1 kPa) (Cesarz and Tamama, 2016), promoting excessive cell spreading and intracellular tension (Cesarz and Tamama, 2016). Moreover, the lack of vertical support in 2D culture induces unnatural apical-basal polarity, which can disrupt signaling and further influence cell shape and size (Duval et al., 2017).

However, several recent well-designed studies challenge this conventional view and suggest that cell size enlargement may lead to cellular senescence (Lengefeld et al., 2021; Neurohr et al., 2019). Human cells tend to grow slightly larger over their lifespans because, during each division, they pause to check for DNA damage. If damage is detected, the cell halts division to make repairs. These delays allow the cell to grow incrementally larger. Over time, with repeated divisions and repair pauses, cells accumulate size. As cells enlarge, their DNA and protein synthesis machinery struggle to meet the demands of the increased volume. This imbalance leads to insufficient protein production, resulting in cytoplasmic dilution and disruption of normal cell division. Diluted cytoplasm slows down biochemical reaction rates, as lower concentrations of key proteins may prevent certain reactions from occurring altogether. Based on this emerging theory, strategies that reduce cell size may help delay the onset of senescence.

### 3D spheroid culture reduces cell size and prolongs lifespan

Studies suggest that 3D spheroid culture can significantly reduce MSC size (Cesarz and Tamama, 2016; Ohori-Morita et al., 2025; Sun et al., 2018; Fuentes et al., 2022). This reduction in cell size enhances the functional properties of MSCs, including increased secretion of therapeutic factors and improved survival after transplantation. The size reduction in 3D spheroids may be driven by multiple factors, including cytoskeletal reorganization, a shift from cell-ECM to cell-cell adhesion, the softer mechanical environment of spheroids, and metabolic constraints resulting from limited nutrient diffusion (Cesarz and Tamama, 2016). A recent study suggests that the size decrease is attributed to an increase in the excretion of extracellular vesicles (Mo et al., 2018). They found that 3D culture led to more vesicles both on the cell surface and in the surrounding medium. This increased shedding appears linked to lower levels of F-actin, a component of the cell’s internal skeleton, suggesting that reducing internal tension facilitates vesicle release and subsequent cell size reduction. However, the molecular mechanisms underlying cell size reduction in spheroid culture remain inconclusive and require further investigation.

While spheroid culture offers many benefits, it is not suitable for large-scale expansion on its own due to the limited proliferative capacity of MSCs in this format. To address this challenge, a recent study proposed a cyclical aggregation strategy that alternates between 3D spheroid formation and 2D monolayer expansion (Cesarz and Tamama, 2016). This approach successfully preserved MSC morphology (spindle shape), proliferation, clonogenicity, differentiation potential, and expression of stemness markers (Nanog, Sox2) over 15 passages tested, while maintaining minimal expression of the senescence-associated protein β-galactosidase. In contrast, MSCs cultured continuously in 2D conditions exhibited significantly reduced proliferation, clonogenicity, differentiation capacity, and expression of stemness markers by passage 15, along with elevated β-galactosidase levels compared to passage five cells. The study also identified activation of the integrated stress response (ISR) pathway as a key mechanism underlying the beneficial effects of the cyclical aggregation strategy. However, the investigation was limited to adipose-derived MSCs, and its applicability to MSCs from other tissue sources remains uncertain. Furthermore, the study did not evaluate whether cyclical aggregation can reduce cell size and enhance functional properties.

### The importance of placenta-derived MSCs

Among various tissue sources, the placenta is particularly attractive for MSC isolation due to its abundance and the large number of MSCs that can be harvested from a single placenta. Over the past decade, our team has focused on using placenta-derived MSCs to treat a range of conditions, with a particular emphasis on myelomeningocele (MMC), or spina bifida, which is caused by incomplete neural tube closure during spinal cord development (Vanover et al., 2019; Yamashiro et al., 2020; Sto et al., 2022; Jackson et al., 2021; Kabagambe et al., 2018; Theodorou et al., 2022a; Clark et al., 2019; Stokes et al., 2021; Pan et al., 2023; Chen et al., 2018; Theodorou et al., 2022b; Zhang et al., 2021; Stokes et al., 2022; Long et al., 2019; Prins Henk-Jan, 2014; Lankford et al., 2017; Galganski et al., 2020). Intrauterine damage to the exposed spinal cord leads to lifelong paralysis, incontinence, musculoskeletal deformities, and cognitive impairments. Our *in vitro* studies demonstrated that placenta MSCs protected neurons from various insults (Sto et al., 2022). Using a retinoic acid (RA)-induced fetal rat MMC model, we found that *in utero* treatment with placenta MSCs significantly reduced spinal cord compression and neuronal apoptosis (Chen et al., 2018). We validated these results in a fetal lamb model of MMC (Vanover et al., 2019; Yamashiro et al., 2020; Sto et al., 2022; Jackson et al., 2021; Kabagambe et al., 2018; Theodorou et al., 2022a; Theodorou et al., 2022b; Stokes et al., 2022; Prins Henk-Jan, 2014; Galganski et al., 2020). Based on these findings, the Phase 1 Cellular Therapy for *In Utero* Repair of Myelomeningocele (CuRe) Trial was started in spring 2021 with seven patients. In 2023, a Phase 2 trial was launched to enroll 28 more patients to assess efficacy after the FDA and the Data Monitoring Board determined that the therapy is safe.

Throughout our journey with the CuRe trial, we have encountered all the MSC culture challenges. While we are currently able to produce sufficient low-passage, functional MSCs using 2D flasks for this proof-of-concept Phase 1/2a trial, involving just 35 patients and a modest dose of 1 × 10^6^ MSCs per patient, existing technologies are inadequate to meet the demands of broader clinical applications following FDA approval. This limitation becomes even more critical when considering treatment for prevalent conditions such as stroke or myocardial infarction, which affect millions and require substantially higher cell doses (∼10^9^ MSCs per patient). There is an urgent need for transformative MSC culture technologies to unlock the full therapeutic potential of MSCs.

### A potential scalable strategy for leveraging the alternating 2D/3D culture approach

While our results show that the alternating 2D/3D approach can significantly improve MSC culture, the protocol is difficult to implement at large scales. Adding a 3D spheroid culture significantly increases the complexity and cost of the process. New culture systems that allow for both 2D and 3D cultures would facilitate the implementation of this new approach. This need might be met with the hydrogel tube microbioreactors recently developed by our lab (Wang and Lei, 2019; Wang et al., 2021; Yang et al., 2025; Lin et al., 2019a; Li et al., 2018a; Lin et al., 2018a; Lin et al., 2019b; Liu et al., 2023; Li et al., 2018b; Lin et al., 2018b; Lin et al., 2019c). This method cultivates cells in hollow, microscale alginate hydrogel tubes. AlgTubes offer a cell-friendly microenvironment, leading to paradigm-shifting improvements in cell viability, growth rate, yield, culture consistency, and scalability. When culturing human pluripotent stem cells, we achieved up to 4000-fold expansion per passage and 5 × 10^8^ cells/mL volumetric yield, which is ∼250 times the current state-of-the-art. However, AlgTubes do not support the growth of anchor-dependent stem cells, as they lack adhesion points.

**FIGURE 7:**
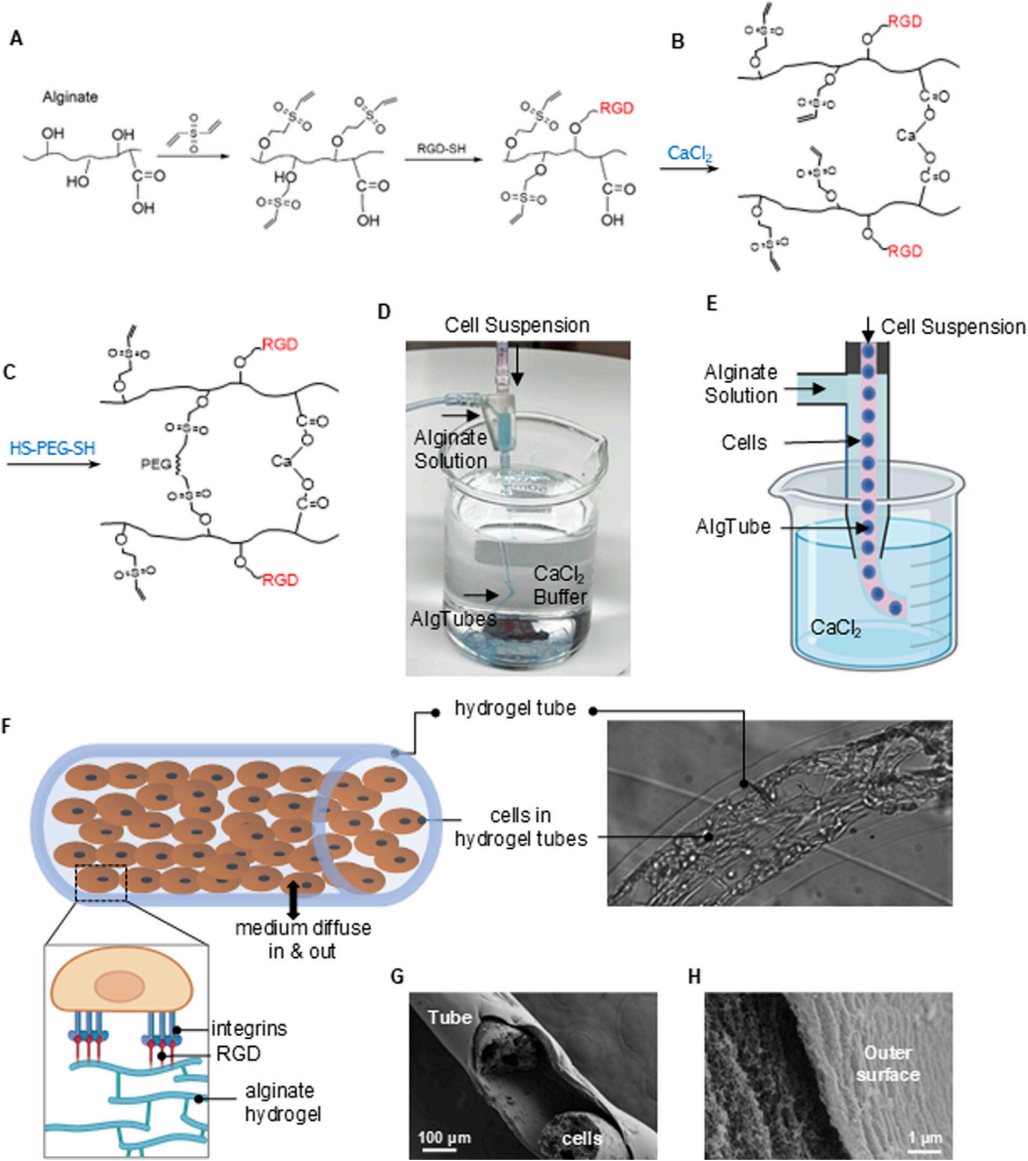
RGD-Modified Alginate Hydrogel Tubes (AlgTubes) for MSC Culture. **(A)** Schematic of alginate modification with RGD peptides. **(B,C)** Chemistry underlying hydrogel tube formation. **(D,E)** Process AlgTubes using a micro-extruder: a cell suspension and alginate solution are pumped into the central and side channels, respectively, creating coaxial core-shell flows. These are extruded through a nozzle into a CaCl_2_ buffer, where Ca^2+^ ions crosslink the outer alginate shell, forming hydrogel tubes instantly. **(F)** Illustration of growing MSCs within an AlgTube. **(G,H)** SEM images showing the porous structure of the AlgTubes.

To address this challenge, we have developed a chemical strategy to functionalize AlgTubes with peptides containing the Arginyl-Glycyl-Aspartic acid (RGD) motif, which binds integrin receptors, in preliminary studies. Briefly, alginates were reacted with Divinyl Sulfone (DVS). About 30% of the OH groups were conjugated with vinyl sulfones (-VS) (Figure 7A). RGD peptides with a thiol (-SH) group at the C-terminal were then reacted with alginate-VS to prepare RGD-modified alginates (RGD-alginate). The RGD/VS ratio controls the degree of RGD modification. Alginate-RGD and unmodified alginate were mixed and extruded into a CaCl_2_ buffer, where they were rapidly crosslinked to form hydrogel tubes through ionic interactions between Ca^2+^ ions and the carboxyl groups on the alginate chains (Figure 7B). The final RGD concentration in the AlgTubes was controlled by the proportion of alginate-RGD used in the mixture. The AlgTubes were then soaked in poly (ethylene glycol) dithiol (HS-PEG-SH, Mw 3400) to achieve secondary covalent crosslinking via a Michael addition reaction between–SH and–VS groups (Figure 7C). This chemistry is simple, cost-effective, biocompatible, and scalable.

We have also designed a micro-extruder to fabricate alginate hydrogel tubes and load cells (Figures 7D,E). The RGD peptides enabled cells to adhere to the inner surface of the tubes, while the hydrogel provided a soft substrate for cell attachment (Figure 7F). These hydrogel tubes have abundant nanopores that allow nutrients and growth factors to enter the tubes, supporting cell viability and growth (Figures 7G,H).

**FIGURE 8:**
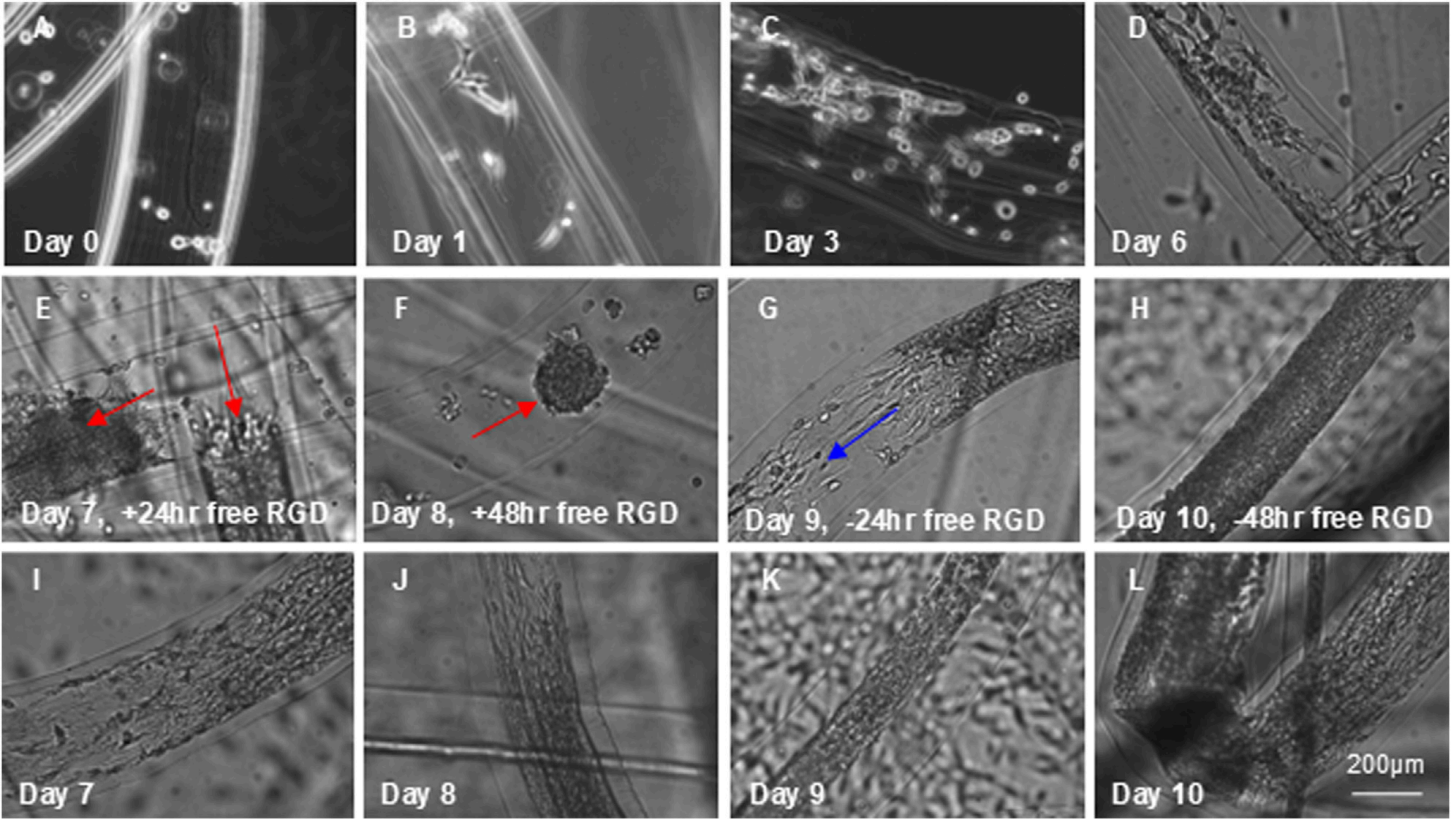
Dynamic Cell Adhesion in RGD-Modified AlgTubes. **(A)** MSCs were processed into RGD-modified AlgTubes. **(B–D)** Cells adhered to the inner surface and proliferated from day 0 to day 6. **(E)** On day 6, free RGD peptides were added to the culture medium, leading to cell contraction within 24 h. **(F)** By 48 h, cells had fully detached and formed spheroids (red arrows). **(G,H)** Removal of free RGD peptides from the medium resulted in MSC reattachment to the AlgTube surface and resumed cell growth (blue arrows). **(I–L)** As a comparison, in the absence of free RGD peptides, day 6 MSCs continued to grow and eventually covered the inner surface of the hydrogel tube.

We then developed a method to implement an alternating 2D/3D culture protocol using RGD-functionalized AlgTubes. MSCs were cultured in these tubes, forming a monolayer along the inner surface (Figures 8A–D), representing the 2D phase. Free RGD peptides were then added to the medium to competitively inhibit integrin receptors, prompting MSCs to contract within 24 h and form spheroids by 48 h (Figures 8E,F), marking the 3D culture phase. Upon removal of free RGD peptides, MSCs reattached and spread, forming a multilayered cell mass within 48 h (Figures 8G,H), thereby reentering the 2D phase. In contrast, MSCs cultured without free RGD peptides remained attached to the hydrogel tube and continued to grow (Figures 8I–L). These results demonstrate that the AlgTube system enables the alternating 2D/3D culture strategy without the need for passaging or changing culture vessels. However, this work is preliminary, and further studies are needed to optimize the system and systematically characterize MSCs.

### Limitations of the study

The present study effectively demonstrated the potential of the alternating 2D/3D culture technique. However, the molecular mechanisms underlying its benefits remain unexplored and warrant further investigation. Insights from existing literature may help illuminate these mechanisms. For instance, one study compared various scaffold-free 3D culture techniques—including micro-well, hanging drop, and ultra-low attachment plate-based spheroid culture—to traditional 2D Culture in Wharton’s jelly-derived MSCs (Thakur et al., 2022). Spheroid Culture significantly enhanced the expression of growth and immunomodulatory factors such as indoleamine 2,3-dioxygenase (IDO), interleukin-10 (IL-10), leukemia inhibitory factor (LIF), angiotensin 1 (ANG1), and vascular endothelial growth factor (VEGF), along with pluripotency markers OCT4, SOX2, and NANOG (Thakur et al., 2022). Additionally, spheroid-cultured WJ-MSCs exhibited a markedly increased differentiation potential toward adipogenic, osteogenic, and definitive endodermal lineages (Thakur et al., 2022).

A more recent study found that a 60-h spheroid culture following 2D expansion significantly reduced MSC size (Lu et al., 2024). Unlike their 2D counterparts, these cells showed minimal lung entrapment and were preferentially recruited to skin lesions following intravenous administration. Single-cell RNA sequencing (scRNA-seq) revealed that spheroid culture synchronized the MSCs into a more homogeneous population, reducing the number of subpopulations from six in 2D culture to two in spheroids (Lu et al., 2024). Both scRNA-seq and bulk RNA-seq analyses demonstrated consistently elevated expression of key immunosuppressive factors in spheroid MSCs, including stanniocalcin-1 (STC1), tumor necrosis factor-stimulated gene 6 (TNFAIP6/TSG6), prostaglandin-endoperoxide synthase 2 (PTGS2), interleukin-6 (IL-6), and transforming growth factor-beta (TGF-β). Growth factors such as VEGFA, FGF2, LIF, HGF, and GDNF were also upregulated (Lu et al., 2024). Moreover, spheroid culture appeared to reprogram MSCs toward a more youthful state, as evidenced by increased expression of anti-aging genes such as PTGFRN and SERPINF1 (Lu et al., 2024). Gene ontology analysis revealed upregulation of genes associated with the proteinaceous extracellular matrix (ECM). Detailed gene expression profiling showed a progressive decline in specific collagen components (COL6A3, COL1A1, COL4A1, COL4A2) during the transition from 2D to spheroid Culture. This reduction in collagen expression was inversely correlated with the upregulation of secretory immunosuppressive factors, such as IL-6, IL-11, and PTGS2 (Lu et al., 2024). The reprogramming of MSCs toward a secretory immunosuppressive phenotype in 3D culture appears to be mediated by ECM remodeling and activation of the AP-1 transcriptional complex. A transient increase in AP-1 components (FOS, JUN, JUNB) linked the downregulation of collagen genes to the upregulation of immunosuppressive factors. These findings suggest that AP-1 plays a central role in mediating the enhanced immunomodulatory profile of MSCs in spheroid culture. It is plausible that similar molecular mechanisms are at play in the alternating 2D/3D culture strategy. Future studies should aim to confirm this hypothesis and further elucidate the underlying pathways.

## Conclusion

Alternating 2D and 3D spheroid Culture effectively mitigates enlargement and senescence of placental MSCs, preserving their proliferative and immunomodulatory functions. MSCs cultured using this approach maintain a smaller size and exhibit enhanced therapeutic potential compared to conventionally expanded 2D cultures. RGD-functionalized AlgTubes offer a scalable platform for implementing this method. Further studies are needed to elucidate the molecular and functional changes induced by this novel technique.

## Data Availability

The original contributions presented in the study are included in the article/Supplementary Material, further inquiries can be directed to the corresponding authors.
