# Supplementary material for: Alternating 2D and 3D culture reduces cell size and extends the lifespan of placenta-derived mesenchymal stem cells
Source: Front Bioeng Biotechnol. 2025 Aug 22;13:1632810. doi: 10.3389/fbioe.2025.1632810 (PMC12411557; doi:10.3389/fbioe.2025.1632810)
Supplement: Supplementary file 1 [file Presentation1.pptx]

## Slide 1
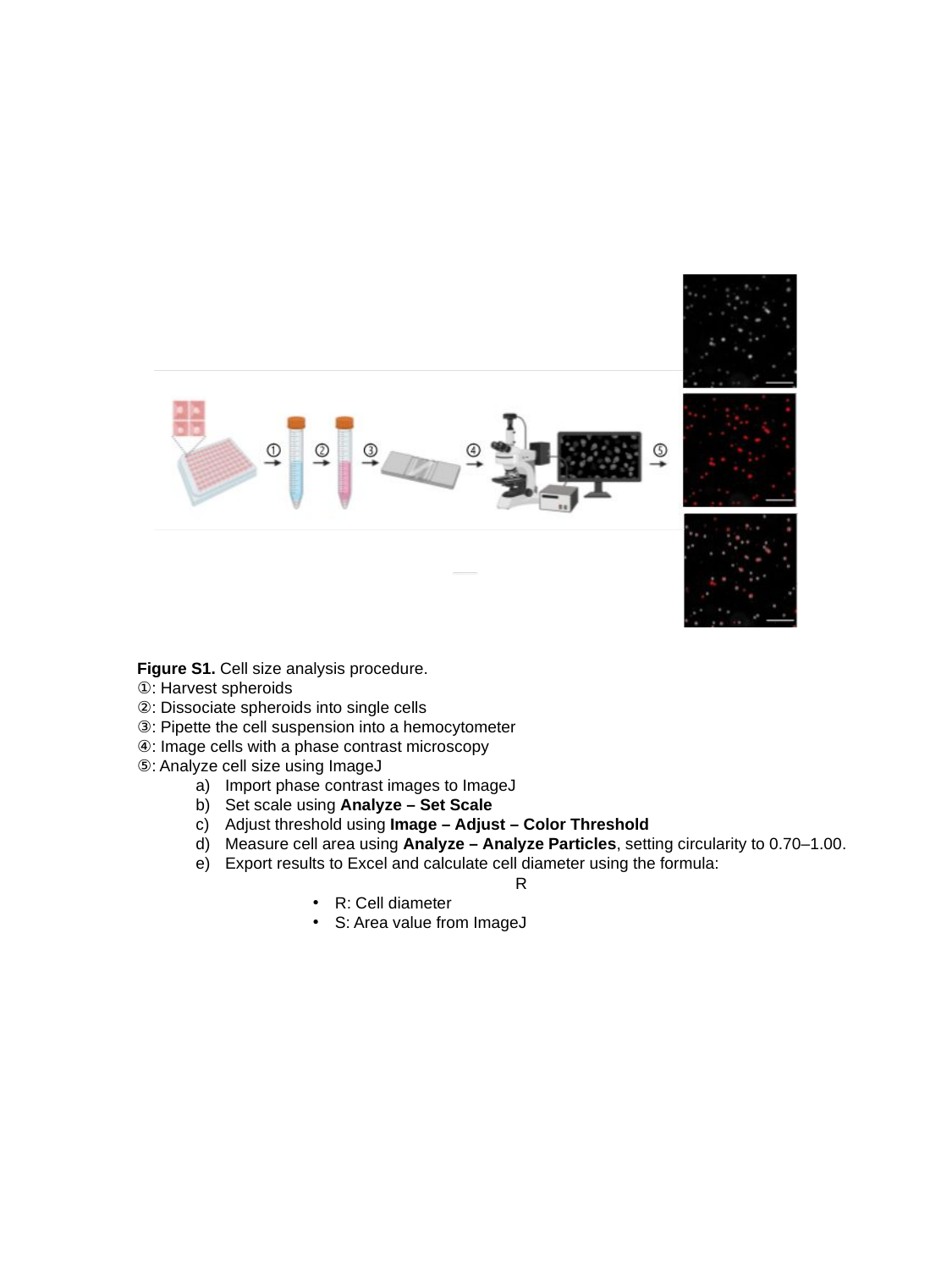

## Slide 2
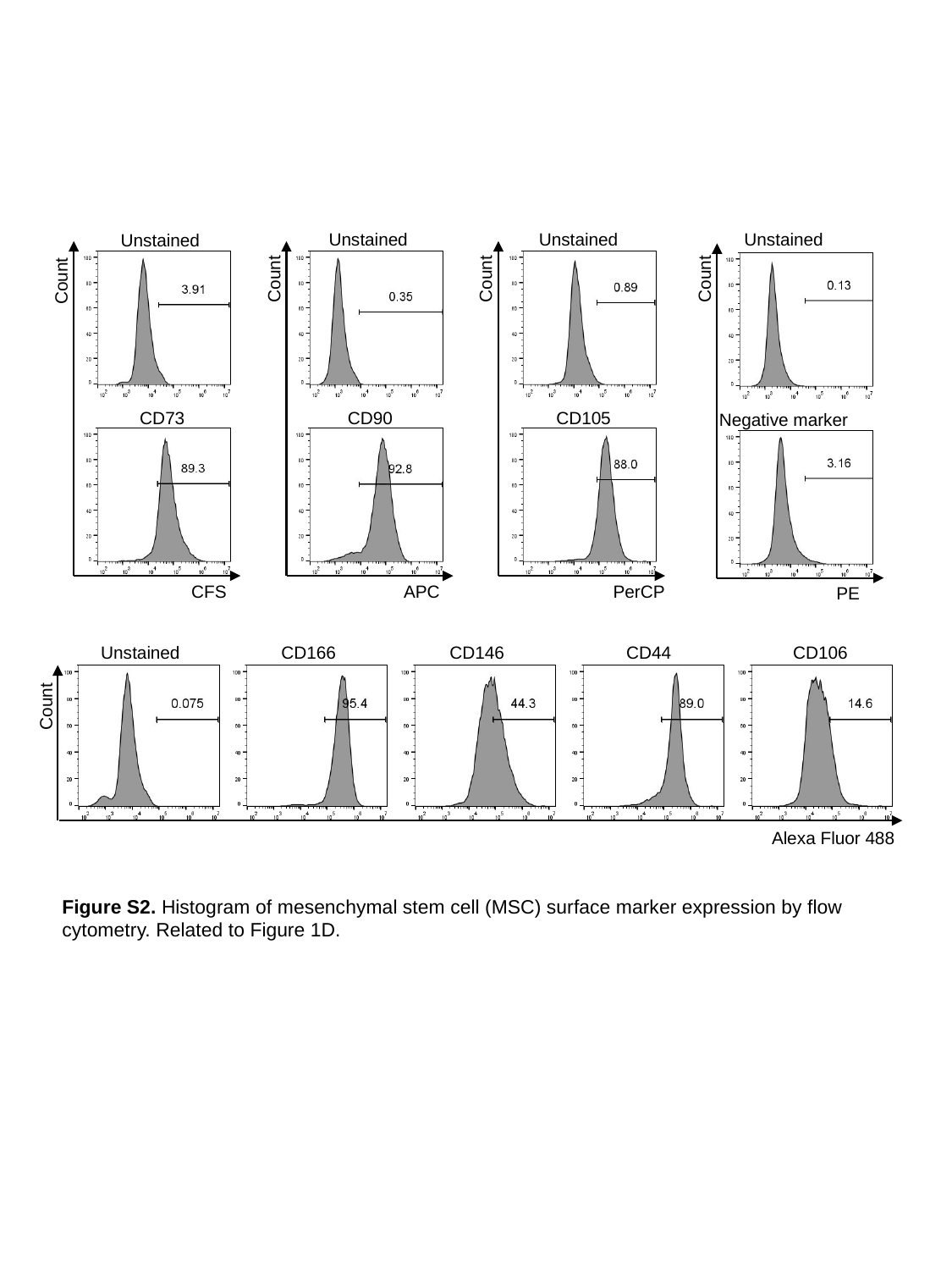

Unstained
Count
APC
CD90
Unstained
Count
PE
Negative marker
Unstained
Count
PerCP
CD105
Unstained
Count
CFS
CD73
Unstained
CD166
CD146
CD44
CD106
Count
Alexa Fluor 488
Figure S2. Histogram of mesenchymal stem cell (MSC) surface marker expression by flow cytometry. Related to Figure 1D.

## Slide 3
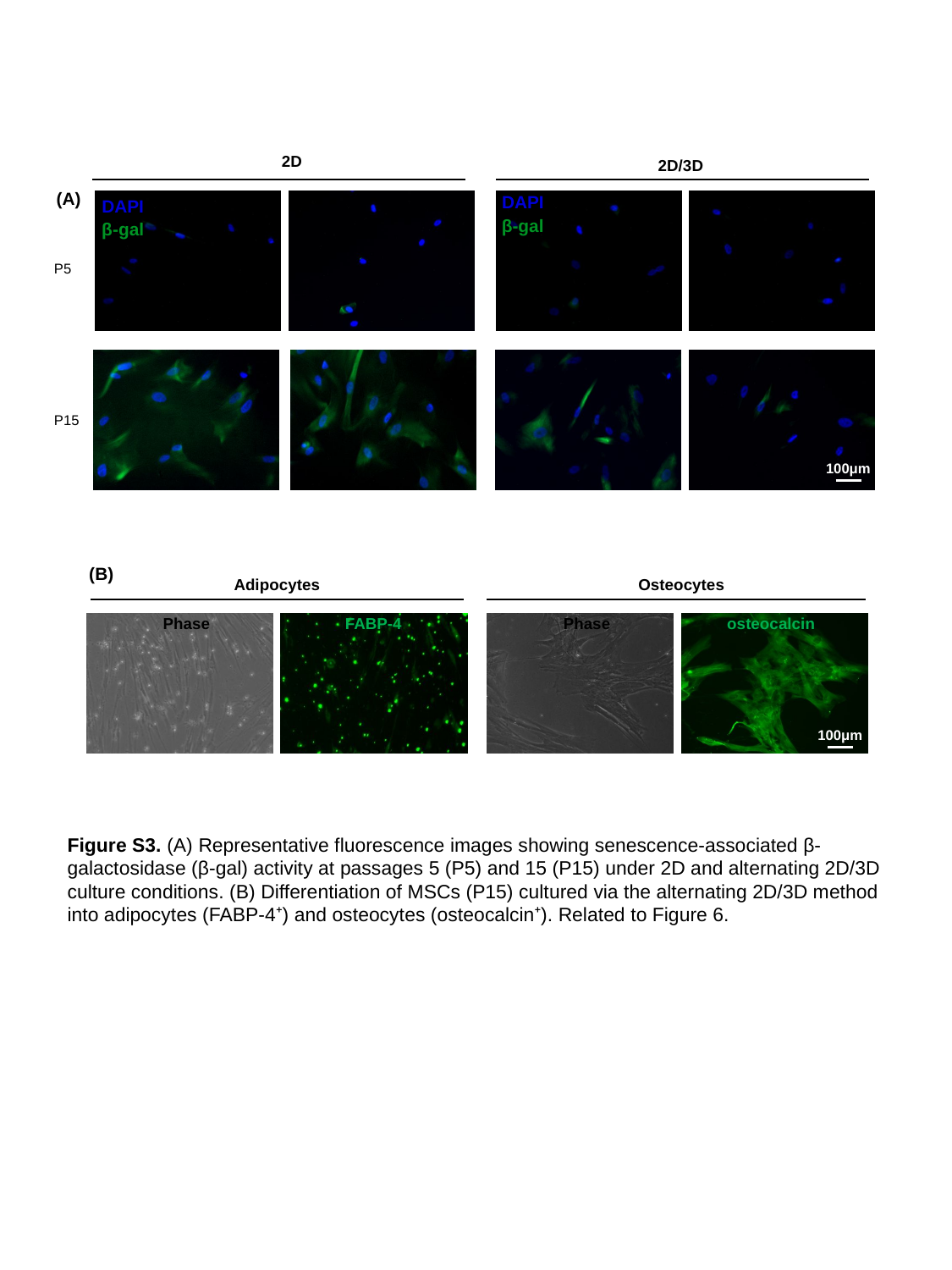

2D
2D/3D
DAPI
β-gal
DAPI
β-gal
P5
P15
100μm
(A)
(B)
Adipocytes
Osteocytes
Phase
Phase
osteocalcin
FABP-4
100μm
Figure S3. (A) Representative fluorescence images showing senescence-associated β-galactosidase (β-gal) activity at passages 5 (P5) and 15 (P15) under 2D and alternating 2D/3D culture conditions. (B) Differentiation of MSCs (P15) cultured via the alternating 2D/3D method into adipocytes (FABP-4⁺) and osteocytes (osteocalcin⁺). Related to Figure 6.
